# Gynandroblastoma With a Juvenile Granulosa Cell Tumor Component Presenting as Ovarian Torsion in a 14-Year-Old Female Patient

**DOI:** 10.7759/cureus.97068

**Published:** 2025-11-17

**Authors:** Lisa Su, Jasmine N Jefferson, Leslie Lopez-Calderon

**Affiliations:** 1 Pathology and Laboratory Medicine, The University of Tennessee Health Science Center, Memphis, USA

**Keywords:** dicer mutation, gynandroblastoma, juvenile granulosa cell tumor, mixed sex cord-stromal tumor, ovarian torsion, ovarian tumor

## Abstract

We report a rare case of a 14-year-old female who presented with a left adnexal mass suspected to be torsed based on ultrasonographic findings. She underwent a left salpingo-oophorectomy, and histopathologic evaluation revealed a mixed sex cord stromal tumor, specifically a gynandroblastoma composed of a juvenile granulosa cell tumor and a moderately differentiated Sertoli-Leydig cell tumor (SLCT) component. Although extremely uncommon, gynandroblastoma should be considered in the differential diagnosis when encountering diverse tumor morphology in ovarian masses. The histologic grade of the SLCT component may influence prognosis and should be closely evaluated.

## Introduction

Gynandroblastoma is an uncommon type of ovarian mixed sex cord-stromal tumor histologically composed of both female and male sex cord differentiation [1]. The male component consists of a Sertoli cell tumor or a Sertoli-Leydig cell tumor (SLCT), while the female component consists of a granulosa cell tumor (GCT) of either juvenile (JGCT) or adult-type (AGCT). Gynandroblastoma with a JGCT component is particularly rare, with only 10 such cases reported in the English-language literature to date [2-11]. For a diagnosis of gynandroblastoma, both components should be recognizable and present in substantial proportions, established by Scully & Young in 1973 to be at least 10% of the total tumor volume [12], although this threshold is not explicitly defined in the 2020 World Health Organization classification [1]. Here, we present a rare case of a 14-year-old female patient with a large unilateral ovarian mass diagnosed as gynandroblastoma with JGCT and moderately differentiated SLCT components, found to harbor a pathogenic germline DICER1 mutation.

## Case presentation

A 14-year-old girl presented to the emergency department with vague abdominal pain for several weeks, which became localized to the left lower quadrant one week prior to admission. Pelvic ultrasonography revealed a large, predominantly solid left adnexal mass measuring 16 × 12 × 10 cm with suspicion of torsion.

The patient had no significant past medical history or known genetic syndromes. She had experienced menarche at age 10, with irregular menses since, but no signs or symptoms of virilization or other endocrine disturbances. Preoperative serum markers showed elevated levels of lactase dehydrogenase (LDH) (480 U/L), cancer antigen 125 (CA-125) (93.2 U/mL), and inhibin B (1,453.1 pg/mL), as shown in Table 1. Alpha-fetoprotein (AFP) (35.3 ng/mL) was at the upper limit of normal. Levels of carcinoembryonic antigen (CEA) and CA 19-9 were within normal limits. Based on the working diagnosis of ovarian torsion with a possible dermoid cyst, the patient underwent a left salpingo-oophorectomy.

**Table 1:** Serum tumor marker laboratory values CA-125, cancer antigen 125; CA 19-9, carbohydrate antigen 19-9; CEA, carcinoembryonic antigen; AFP, alpha-fetoprotein; LDH, lactase dehydrogenase

Tumor Marker	Serum Level (Reference Range)
CA-125	93.2 (0.0-35.0 U/mL)
CA 19-9	14 (0-35 U/mL)
CEA	<1.7 (0.0-5.0 ng/mL)
AFP	35.3 (0.8-34.8 ng/mL)
LDH	480 (174-258 U/L)
Inhibin B	1,453.1 (<362.0 pg/mL)

**Figure 1:**
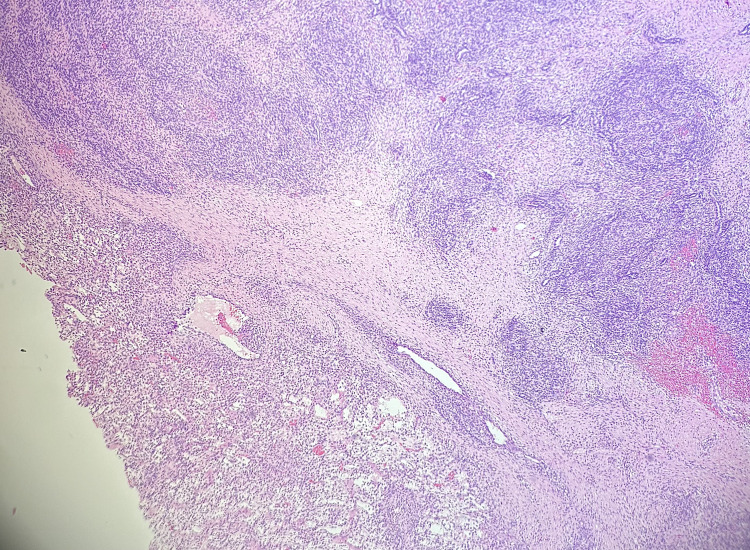
Mixed Sertoli-Leydig cell tumor (upper right) and juvenile granulosa cell tumor (lower left), H&E 4x

**Figure 2:**
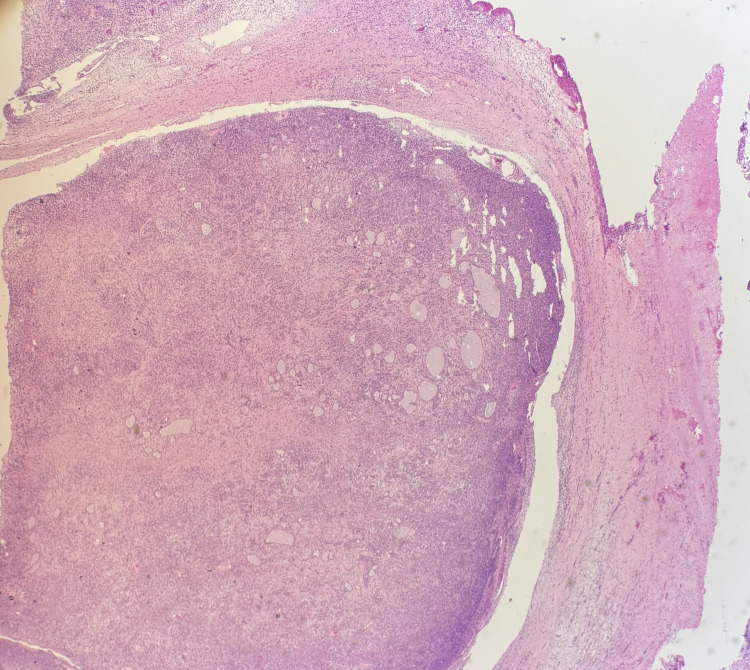
Juvenile granulosa cell tumor shows solid and nodular growth patterns with relatively uniform tumor cells, H&E 2x

**Figure 3:**
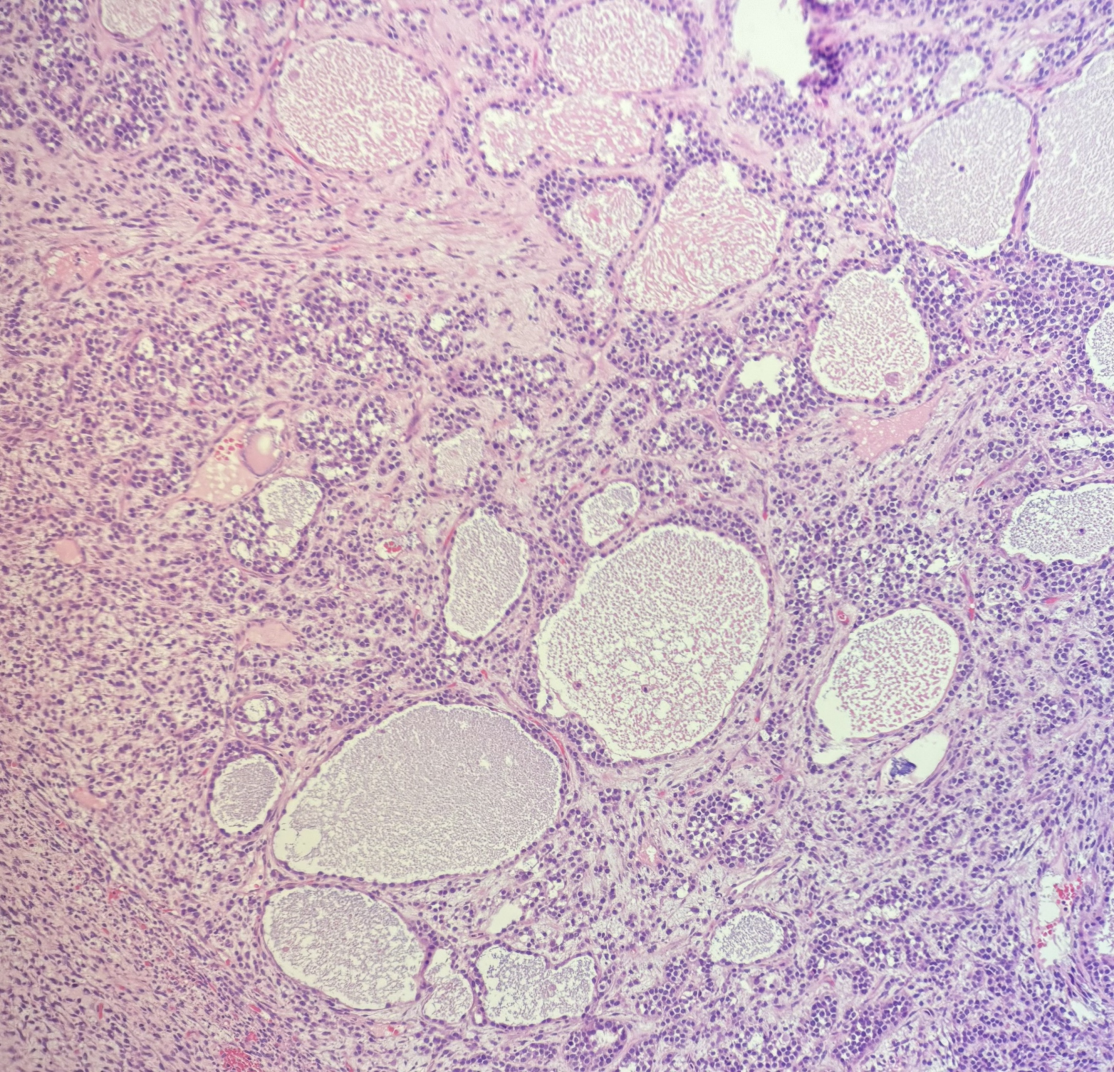
Juvenile granulosa cell tumor with variably sized follicle-like spaces containing basophilic secretions, H&E 4x

**Figure 4:**
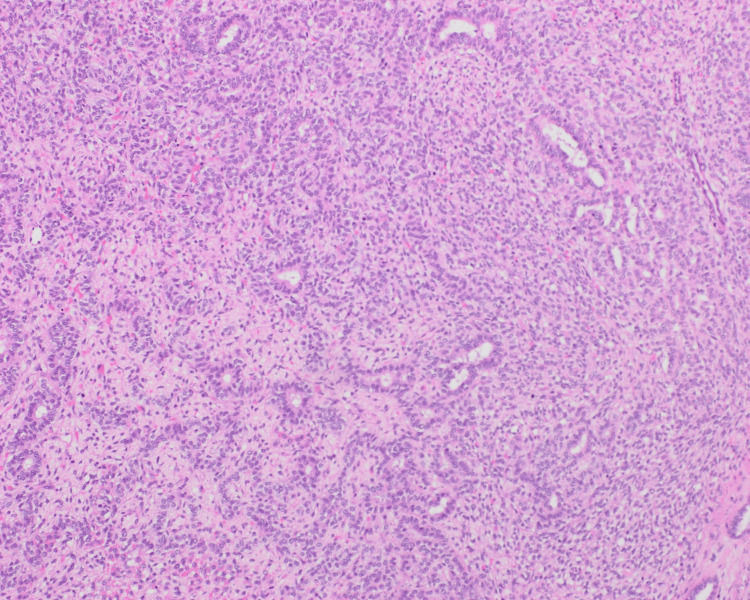
Focal areas of the well-differentiated Sertoli-Leydig cell tumor with tubular differentiation admixed with Leydig cells, H&E 10x

**Figure 5:**
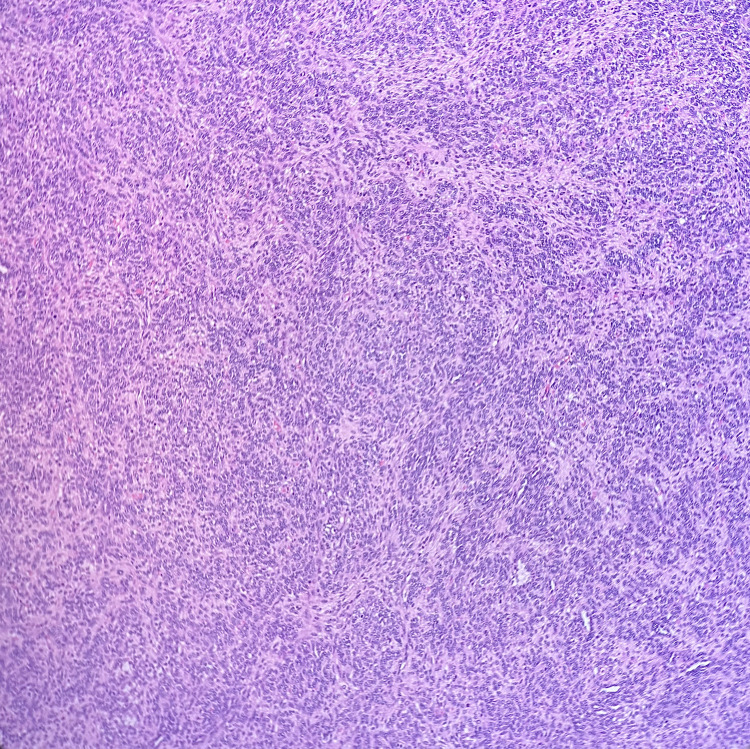
Sertoli-Leydig cell tumor with intermediate differentiation and spindle cell areas without tubular formation seen, H&E 10x

Gross examination revealed a 20.5 × 14.2 × 14.0 cm solid, yellowish, variegated mass confined to the ovary with an intact capsule. Microscopically, the tumor displayed nodular and diffuse growth patterns comprising two main components (Figure 1). A JGCT component involved 30-40% of the total tumor volume and was characterized by lobulated architecture with follicle-like and cystic spaces, round hyperchromatic nuclei, and eosinophilic to clear cytoplasm (Figure 2). Macro- and microfollicles with basophilic secretions were observed (Figure 3). A second moderately differentiated SLCT component involving 60-70% of the tumor consisted of ovoid tumor cells with hyperchromatic nuclei, small nucleoli, and amphophilic cytoplasm, arranged in cords and trabeculae (Figures 4, 5). Focal hemorrhagic infarction and well-differentiated areas were present.

**Figure 6:**
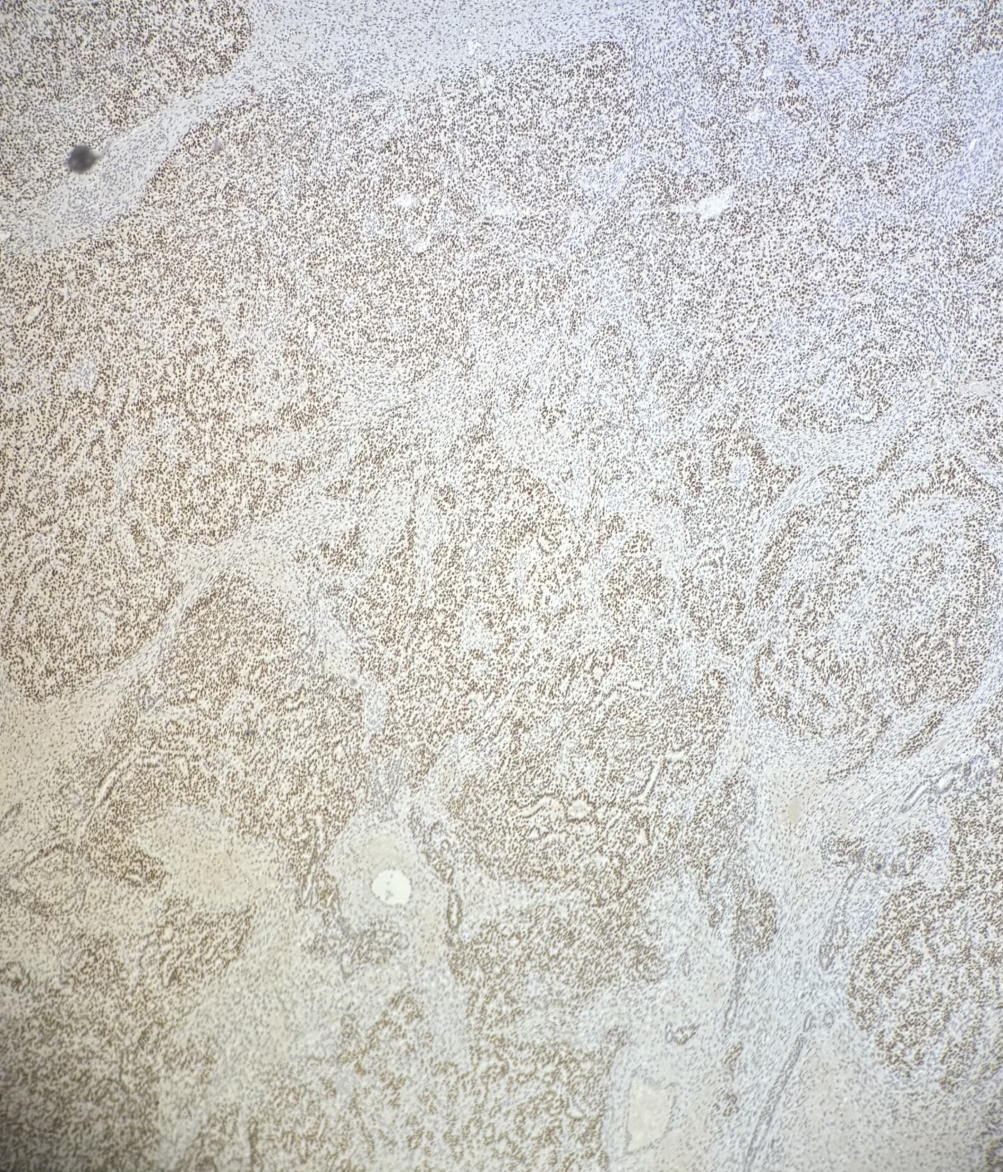
Both components stained positive for SF-1, confirming that the tumor was of sex cord-stromal origin

**Figure 7:**
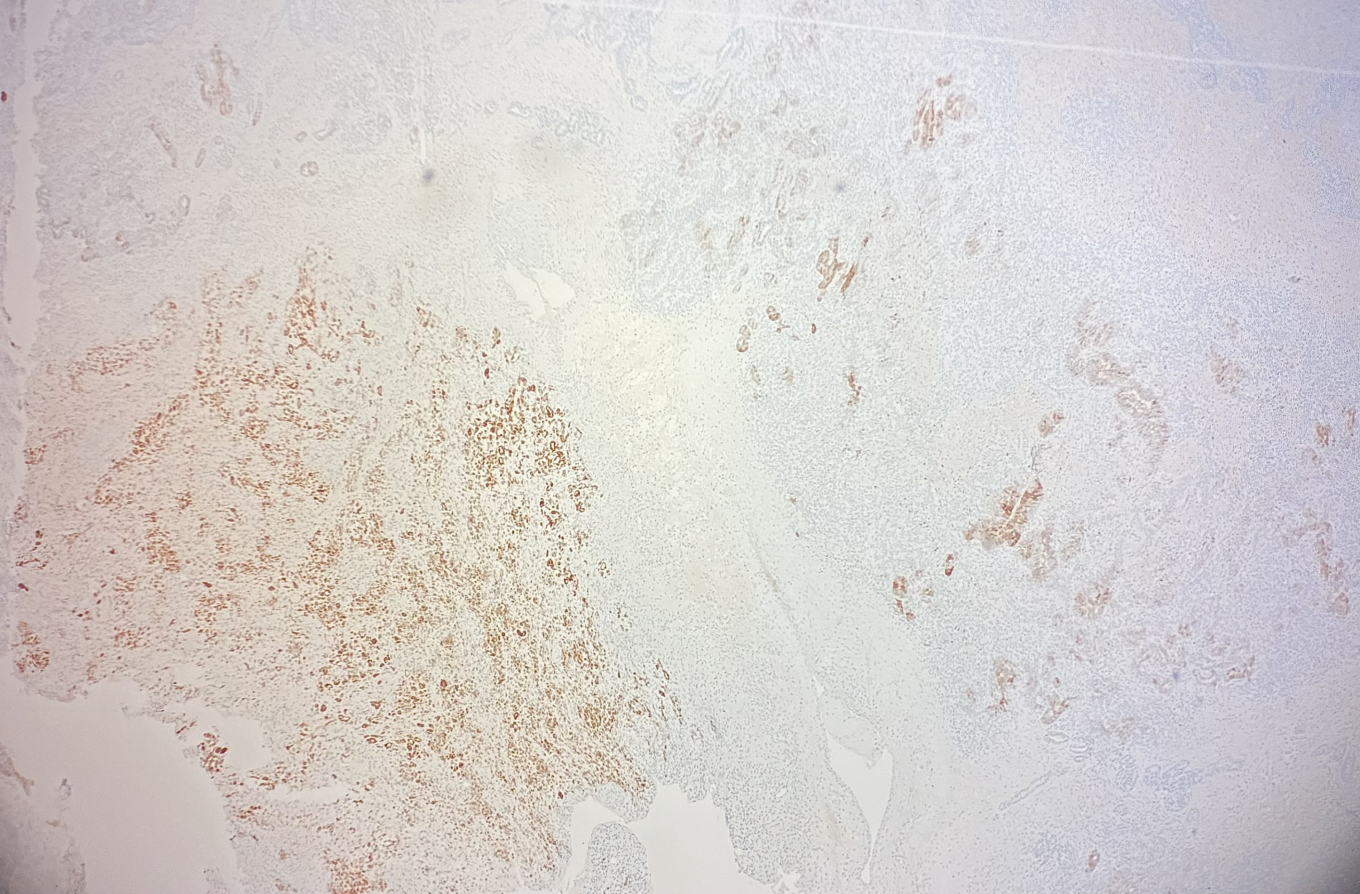
Both components stained positive for calretinin, confirming that the tumor was of sex cord-stromal origin

**Figure 8:**
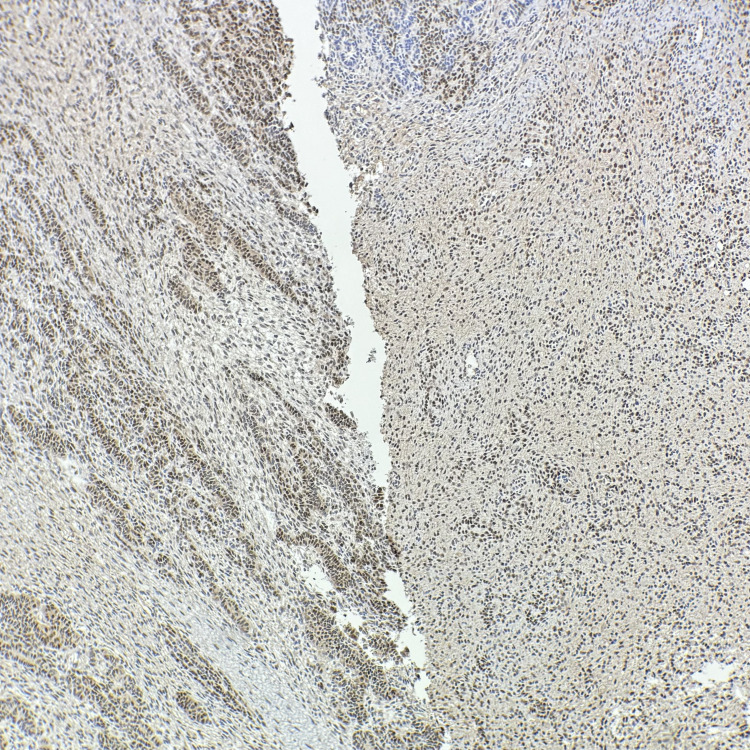
Both components stained positive for FOXL2, confirming that the tumor was of sex cord-stromal origin

Immunohistochemistry (IHC) showed both components were positive for SF-1, calretinin, and FOXL2, and negative for SALL4 (Figures 6-8). Myogenin and MyoD1 were also negative, helping to exclude sarcomatous transformation. Inhibin showed variable positivity in both components. 

Postoperative recovery was uneventful. The tumor was confined to the ovary (FIGO Stage I, 2014 revision) [13], and no adjuvant treatment was required. Molecular analysis revealed a pathogenic germline DICER1 mutation. The patient was subsequently enrolled in a surveillance program for individuals with germline DICER1 mutations and is currently under clinical surveillance.

## Discussion

Gynandroblastoma is a rare type of mixed sex cord-stromal tumor of the ovary, classified alongside SLCTs and sex cord-stromal tumors not otherwise specified under the 2020 World Health Organization framework [1]. Gynandroblastoma with a JGCT occurs most commonly between the second and fourth decades of life [2-9], with the mean age at diagnosis being 28 years [14]. However, there have also been two cases of gynandroblastoma with JGCTs occurring in 65- and 71-year-old patients [10,11]. Most patients present with large unilateral tumors ranging from 10 to 20 cm in size, producing symptoms of abdominal discomfort, distention, or acute pain due to torsion. Endocrine manifestations such as abnormal menstrual bleeding and virilization, especially hirsutism, are common among both adolescents and adults [3,5-9].

Reports in the literature have shown that many cases of gynandroblastoma with JGCTs are limited to the unilateral ovary at the time of diagnosis (FIGO stage Ia) [14], permitting management with fertility-sparing surgery alone if there is no evidence of tumor rupture [15,16]. On the other hand, patients who are perimenopausal or have completed childbearing are typically managed with total hysterectomy and bilateral salpingo-oophorectomy [5,8-10]. Definitive diagnosis of gynandroblastoma relies on histopathological examination of the excised tumor for an admixture of male and female components [1]. Beyond histomorphology, immunohistochemical markers such as SF-1 [6,9], calretinin [6,8,10], inhibin [8-11], and FOXL2 [17,18] are useful in confirming sex cord-stromal lineage for tumors of the ovary.

Our case also emphasizes the growing importance of molecular testing in sex cord-stromal tumors for not only diagnostic support but also identification of germline mutations, which carry implications for prognosis, surveillance, and family counseling. Both germline and somatic mutations in DICER1, a gene encoding an endoribonuclease involved in microRNA processing and gene expression regulation, have been associated with SLCTs and gynandroblastomas with JGCT [16,18,19]. Data from the International Ovarian and Testicular Stromal Tumor (OTST) Registry suggest that such tumors present at a younger age in patients with predisposing germline or mosaic DICER1 mutations compared to those with tumor-limited mutations (16 versus 21 years) [16]. Beyond ovarian sex-cord stromal tumors, pathogenic germline DICER1 variants are associated with benign and malignant tumors of the lung, thyroid, kidney, and central nervous system as part of an autosomal dominant tumor predisposition syndrome [20].

Prognosis for gynandroblastoma is generally favorable, especially when diagnosed at an early stage (FIGO stage Ia) [15]. Although patients with germline and mosaic DICER1 mutations are at risk for metachronous, bilateral SLCTs [8,9,15,16], predisposing DICER1 mutations have been associated with improved recurrence-free and overall survival compared to tumor-limited mutations [16]. On the other hand, poor prognostic factors include preoperative tumor rupture as well as the presence of a poorly differentiated SLCT component, which is associated with heterozygous hotspot mutations in the RNase IIIb domain of DICER1 [15,16,20].

Current consensus guidelines from the International DICER1 Symposium recommend germline testing for all individuals diagnosed with ovarian SLCT or gynandroblastoma [20]. Surveillance recommendations of the female reproductive tract for those with pathogenic germline DICER1 variants include pelvic ultrasound and serum hormone panels beginning around 8-10 years old to 40 years old [20]. Additionally, clinicians should counsel patients on signs of hormonal excess, such as hirsutism or menstrual irregularities, which may indicate tumor development or recurrence.

## Conclusions

This case highlights symptoms of ovarian torsion as the initial presentation of a rare case of ovarian gynandroblastoma and the need for prompt surgical intervention to avoid the potential for rupture, which would upstage the disease and could worsen prognosis. This case also reinforces the critical role of thorough histopathologic evaluation and molecular testing in guiding diagnosis, management, and long-term surveillance. In addition, it underscores the importance of considering rare mixed sex cord-stromal tumors in the differential diagnosis of large adnexal masses in adolescents, even in the absence of endocrine symptoms. Early identification of a germline DICER1 mutation enables appropriate genetic counseling and tailored surveillance strategies for both the patient and at-risk relatives. Broader awareness of these rare entities among clinicians and pathologists can facilitate timely recognition and optimal patient outcomes.

## References

[1] Kommoss F, Karnezis AN (2020). Gynandroblastoma. WHO Classification of Female Genital Tumours.

[2] McCluggage WG, Sloan JM, Murnaghan M, White R (1996). Gynandroblastoma of ovary with juvenile granulosa cell component and heterologous intestinal type glands. Histopathology.

[3] Broshears JR, Roth LM (1997). Gynandroblastoma with elements resembling juvenile granulosa cell tumor. Int J Gynecol Pathol.

[4] Talerman A (1998). Gynandroblastoma with elements of juvenile granulosa cell tumor. Int J Gynecol Pathol.

[5] Chan R, Tucker M, Russell P (2005). Ovarian gynandroblastoma with juvenile granulosa cell component and raised alpha fetoprotein. Pathology.

[6] Wilberger A, Yang B (2015). Gynandroblastoma with juvenile granulosa cell tumor and concurrent renal cell carcinoma: a case report and review of literature. Int J Surg Pathol.

[7] Takeda A, Watanabe K, Hayashi S, Imoto S, Nakamura H (2017). Gynandroblastoma with a juvenile granulosa cell component in an adolescent: case report and literature review. J Pediatr Adolesc Gynecol.

[8] Mercier AM, Zorn KK, Quick CM, Huffman LB (2021). Recurrent gynandroblastoma of the ovary with germline DICER1 mutation: a case report and review of the literature. Gynecol Oncol Rep.

[9] Maraqa B, Al-Ashhab M, Kamal N, Sughayer M, Barakat F (2023). Mixed sex cord-stromal tumor (gynandroblastoma) with malignant morphology involving both ovaries: a case report. J Int Med Res.

[10] Jang NR, Lee DH, Jang EJ, Bae YK, Baek J, Jang MH (2018). Ovarian gynandroblastoma with a juvenile granulosa cell tumor component in a postmenopausal woman: a case report and literature review. J Pathol Transl Med.

[11] Hwang S, Kim BG, Song SY, Kim HS (2020). Ovarian gynandroblastoma with a juvenile granulosa cell tumor component in a postmenopausal woman. Diagnostics (Basel).

[12] Chivukula M, Hunt J, Carter G, Kelley J, Patel M, Kanbour-Shakir A (2007). Recurrent gynandroblastoma of ovary-a case report: a molecular and immunohistochemical analysis. Int J Gynecol Pathol.

[13] Mutch DG, Prat J (2014). 2014 FIGO staging for ovarian, fallopian tube and peritoneal cancer. Gynecol Oncol.

[14] Ordulu Z, Young RH (2021). Sertoli-Leydig cell tumors of the ovary with follicular differentiation often resembling juvenile granulosa cell tumor: a report of 38 cases including comments on sex cord-stromal tumors of mixed forms (so-called gynandroblastoma). Am J Surg Pathol.

[15] Schultz KA, Harris AK, Schneider DT (2016). Ovarian sex cord-stromal tumors. J Oncol Pract.

[16] Schultz KA, Harris AK, Finch M (2017). DICER1-related Sertoli-Leydig cell tumor and gynandroblastoma: clinical and genetic findings from the International Ovarian and Testicular Stromal Tumor Registry. Gynecol Oncol.

[17] Al-Agha OM, Huwait HF, Chow C (2011). FOXL2 is a sensitive and specific marker for sex cord-stromal tumors of the ovary. Am J Surg Pathol.

[18] Wang Y, Karnezis AN, Magrill J (2018). DICER1 hot-spot mutations in ovarian gynandroblastoma. Histopathology.

[19] Heravi-Moussavi A, Anglesio MS, Cheng SW (2012). Recurrent somatic DICER1 mutations in nonepithelial ovarian cancers. N Engl J Med.

[20] Schultz KA, Williams GM, Kamihara J (2018). DICER1 and associated conditions: identification of at-risk individuals and recommended surveillance strategies. Clin Cancer Res.

